# Medical Meetings

**Published:** 1916-01

**Authors:** 


### Medical Meetings.

The Tarrant County Medical Association met in Fort Worth December 3d. The following officers were elected: Dr. C. O. Harper, President; Dr. Jas. J. Richardson, Secretary; Dr. W. R. Thompson, Treasurer.

The North Texas Medical Association met in Fort Worth December 7-8, and enjoyed a most successful meeting. The officers elected were: Dr. C. R. Johnson, of Fort Worth, President; Dr. C. O. Harper, of Fort Worth, Vice-President; Dr. H. Leslie Moore, of Dallas, Secretary; Dr. J. H. McLean, of Fort Worth, Treasurer.

The second annual meeting of the Southwest Medical and Surgical Association was held in El Paso December 10-11. El Paso was selected as the permanent meeting place of the Association.

Dr. Walter G. Kimbrough was elected President of the Denton County Medical Society for the ensuing year. Dr. Hill Rowe was elected Vice-President, and Dr. Ada Kincaid, the only lady member, was elected Secretary-Treasurer.

The Jefferson County Medical Society, which met in Beaumont December 6, elected the following officers for the year 1916: Dr. C. A. Cobb, President; Dr. Tom Selman, Vice-President; Dr. Jos. Phillips, Secretary-Treasurer.

The Kaufman County Medical Society met in Kaufman December 7. The annual reported showed that all debts had been paid and that there was $125 in the treasury.

The Orange County Medical Society met in Orange December 7 and elected officers. Dr. J. E. Reeves was elected President; Dr. A. G. Pearce, Vice-President; Dr. J. P. Hewson, Secretary.

The annual election of the officers of the Gonzales County Medical Society was held December 6, with the following results: Dr. Geo. Holmes, President; Dr. A. P. Parr, Vice-President, and Dr. W. T. Dawe, Secretary-Treasurer.

The Anderson County Medical Society elected the following officers at the last meeting for the year 1916: Dr. R. H. McLeod, President; Dr. R. H. Link, Vice-President; Dr. E. B. Parson, Secretary-Treasurer.

New officers were elected by the Falls County Medical Association on December 23, as follows: Dr. F. H. Shaw, President; Dr. W. H. Allen, Vice-President; Dr. A. J. Streit, Secretary.

The Cameron County Medical Society met at Brownsville. Officers were elected and resolutions were adopted to the memory of the late Dr. E. S. McCain. Dr. B. O. Works was elected President; Dr. E. E. Dickinson, Vice-President; Dr. Lawrence was re-elected Secretary and Treasurer. Dr. H. K. Loew was elected delegate to the State convention.

The officers for the Wilbarger County Medical Society for the ensuing year are as follows: Dr. H. H. Rhoads, Vernon, President; Dr. Tom King, Harrold, Vice-President; Dr. R. W. Hix, Vernon, Secretary.

Dr. F. C. Walsh, of San Antonio, was elected President of the County Medical Association for the coming pear; Dr I. P. Nixon was chosen Vice-President; Dr. W. H. Hargis, Secretary; Dr. L. B. Jackson, Treasurer; Dr. B. F. Stout, Censor.

At a meeting of the Bastrop County Medical Society held at Bastrop the following officers were elected: President, Dr. Krowlik; Vice-President, Dr. N. B. Harris; Secretary and Treasurer,

Dr. Carter; Censor, Dr. Chapman; delegate to State Association, Dr. Carter; Alternate, Dr. H. B. Combs.

The Titus County Medical Society at their regular monthly meeting held Tuesday elected officers as follows: W. R. K. Johnson, President; S. R. Crabtree, Vice-President; W. H. Blythe, Secretary-Treasurer; Censors, Drs. D. M. Leftwich, S. R. Crabtree and S. E. Broadstreet. Public Health and Legislation Committee, Drs. Thos. R. Grissom, D. M. Leftwich and Jno. S. Taylor.

The new officers of the Milam County Medical Society are: Dr. G. B. Taylor, President; Dr. W. R. Newton, Vice-President; Dr. Elbra Monroe, Secretary and Treasurer. Dr. Coulter of Rockdale, and Dr. L. L. Lee, of Thorndale, were elected new censors. Dr. Reshier was elected delegate to the State meeting, and Dr. A. S. Epperson, Alternate.

The Denton County Medical Association met at the courthouse Friday evening at 7:30 o'clock for the annual election of officers. Dr. Walter Kimbrough was elected President of the Society, with Dr. Hill Rowe, Vice-President, and Dr. Ada Kincaid, Secretary-Treasurer.

Williamson county has chosen the following for officers for the coming year: Dr. J. H. Vaughan, of Liberty Hill, President; Dr. G. W. Foster, of Georgetown, Vice-President; W. G. Pettus, of Georgetown, Secretary and Treasurer; Dr. J. I. Collins, of Taylor, delegate to the State convention; Dr. C. C. Foster, of Granger, Alternate. Dr. W. H. Moses was elected to fill the vacancy on the Board of Censors.

